# Differential Associations of Executive Functions on a Performance Based Test of Functional Abilities in Older Adults

**DOI:** 10.1002/alz70857_105224

**Published:** 2025-12-25

**Authors:** Moira McKniff, Marina Kaplan, Sophia L. Holmqvist, Tania Giovannetti

**Affiliations:** ^1^ Temple University, Philadelphia, PA, USA

## Abstract

**Background:**

Prior research has established the cognitive domain of executive functioning (EF) as being related to everyday functioning in older adults. Though, it is unclear whether specific aspects of EF are implicated in the completion of everyday activities, which often require executive skills (selection of objects, inhibition of competing stimuli, sequencing task steps, goal maintenance). In this study, we investigate whether more sensitive, computer –based measures of EF from the NIH‐toolbox EXAMINER battery were associated with performance on a test of everyday function (the Naturalistic Action Test [NAT]) and if so, which components of EF (switching, working memory, resisting inhibition) are most important for everyday function.

**Method:**

186 older adults (Mage = 73.4, SD = 6.53, 71% healthy cognition) completed the computer‐based NIH EXAMINER battery, which included tests of inhibition (flanker), set‐shifting, and working memory (*N*‐back). The NAT consisted of two tasks (breakfast and lunch) and was scored for subtle, inefficient errors (micro‐errors) and the number of seconds it took to complete each task step (accomplishment rate).

**Result:**

Multiple linear regression analyses revealed that even when accounting for age (p = .489) and years of education (*p* = .893), EXAMINER measures of inhibition (flanker; β = ‐.1.189, p = .018) and working memory (*n*‐back; β = ‐1.693, p = .003) significantly predicted NAT micro‐errors (R2 = .232, F(5, 103)=6.233, *p* < .001). Similarly, inhibition (flanker; β = ‐.850, p = .006) and working memory (*n*‐back; β = ‐.700, p = .040) significantly predicted NAT accomplishment rate, even when controlling for age (p = .262) and education (p = .340, R2 = .270, F(5, 103)=7.633, *p* < .001). Set shifting did not significantly predict either NAT variable (micro‐errors: β = ‐.299, p = .495; accomplishment rate: β = ‐.480, *p* = . 077).

**Conclusion:**

EXAMINER measures of inhibition and working memory consistently predicted speed (accomplishment rate) and accuracy (micro‐errors) on a task of functional performance. When aiming to improve everyday functioning abilities in older adults, it may be more useful to tailor interventions to boost working memory and inhibition of competing stimuli/task demands over the ability to switch between task steps.

